# Improvement of Microstructure and Mechanical Properties of SiC–VC System Obtained by Electroconsolidation

**DOI:** 10.3390/ma18184331

**Published:** 2025-09-16

**Authors:** Vyacheslav Ivzhenko, Edvin Hevorkian, Miroslaw Rucki, Volodymyr Nerubatskyi, Zbigniew Krzysiak, Volodymyr Chyshkala, Jolanta Natalia Latosińska, Waldemar Samociuk, Tadeusz Szumiata, Tamara Kosenchuk, Jacek Caban

**Affiliations:** 1 V. Bakul Institute for Superhard Materials of the National Academy of Sciences of Ukraine, 2 Avtozavodska St., 04074 Kyiv, Ukraine; 2 Department of Mechanical Engineering and Automation, University of Life Sciences in Lublin, 28 Głęboka St., 20-612 Lublin, Poland; 3 Department of Electrical Energetics, Electrical Engineering and Electromechanics, Ukraine State University of Railway Transport, 7 Feuerbach Sq., 61050 Kharkiv, Ukraine; 4 Institute of Mechanical Science, Vilnius Gediminas Technical University, 11 Sauletekio al., LT-10223 Vilnius, Lithuania; 5 Faculty of Production Engineering, University of Life Sciences in Lublin, 28 Głęboka St., 20-612 Lublin, Poland; waldemar.samociuk@up.lublin.pl; 6 Department of Reactor Engineering Materials and Physical Technologies, V. N. Karazin Kharkiv National University, 4 Svobody Sq., 61022 Kharkiv, Ukraine; 7 Faculty of Physics and Astronomy, Adam Mickiewicz University, Uniwersytetu Poznańskiego 2, 61-614 Poznań, Poland; jolanta.latosinska@amu.edu.pl; 8 Faculty of Mechanical Engineering, Casimir Pulaski Radom University, 54 Stasieckiego, 26-600 Radom, Poland; 9 Faculty of Mechanical Engineering, Lublin University of Technology, Nadbystrzycka 36, 20-618 Lublin, Poland; j.caban@pollub.pl

**Keywords:** silicon carbide, vanadium carbide, sintering, porosity, hardness, toughness

## Abstract

This study examines the influence of vanadium carbide (VC) on the physical and mechanical properties of SiC–VC composites fabricated by a modified spark plasma sintering (SPS) method at a uniaxial pressure of 45 MPa. It was found that the addition of 40 wt.% VC into the SiC matrix led to a substantial reduction in porosity from ca. 30% to less than 8.2% and caused enhancement of the properties. Fracture toughness increased from 2.9 to 7.0 MPa·m^1/2^, and hardness rose from 2.9 to 22.6 GPa. In the SiC–VC system, vanadium carbide acted as a grain growth inhibitor and particulate reinforcement. A sintering temperature increase from 1900 °C to 2000 °C resulted in a ~70% improvement in hardness and a ~50% gain in fracture toughness. The results highlighted the critical balance between densification parameters and microstructural stability. Utilization of n-dimensional vector space of material features, Mahalanobis distance, and Pareto trade-off optimization helped to describe the features of the newly obtained composites and to optimize the manufacturing process.

## 1. Introduction

Silicon carbide (SiC) is a ceramic material with universal mechanical, thermal, and electronic properties widely used in automotive and aerospace industries [1]. Even though it is more known as a wide-bandgap *sp*-semiconductor suitable for high-power and high-temperature electronics [2,3], SiC has significant potential for the production of high-temperature-resistant, wear-resistant, and corrosion-resistant materials due to its high hardness, strength, high creep resistance, and significant oxidation resistance [4]. Its crystalline structure consists of alternating layers of silicon (Si) and carbon (C) atoms, with stable bonds between them. This arrangement is fundamental to the construction of a robust and stable structure of SiC, rendering it a material of significant importance within the domain of material science and engineering.

However, SiC ceramics exhibit some challenging drawbacks, including their brittleness and the high temperature required for the sintering process. It is difficult to achieve high densification of SiC with admixtures due to its high covalent bond ratios and the appearance of oxide film on the surface of raw material powder particles [5]. Boehmler and co-authors [6] demonstrated that the conditioning state of the initial powder had a significant impact on the densification and final microstructure of the ceramic, showing that the granulated powder had the most favorable rheological behavior. There are reports indicating that the sintering temperature could be reduced by the addition of another phase, which not only improved densification but also improved the strength and fracture toughness of the SiC-based composite [7]. Some data can be found concerning sintering of SiC materials with no additives, with metal additives, with boron and carbon additives (B_4_C), and with an AlN additive [8]. Ge et al. demonstrated that SiC powder with the addition of 9 wt.% Y_2_O_3_-Er_2_O_3_ could be sintered at 2000 °C, and the as-prepared composite exhibited improved both electrical resistivity and thermal conductivity [9]. Wozniak, with his team [10], evaluated the effect of the addition of Ti_3_AlC_2_—MAX phases on the consolidation of SiC ceramics. They found that the thermal degradation of Ti_3_AlC_2_ resulted in the formation of titanium carbide as the reinforcing phase, allowing for the production of high-density material. Zheng and co-authors [11] reported the fabrication of a dense SiC-based composite with the addition of TaC using solid-state spark plasma sintering (SPS). They obtained a relative density of 98.0%, a fracture toughness of 8.3 ± 0.8 MPa·m^1/2^, and a Vickers hardness of 17.5 ± 0.4 GPa. Das, with collaborators [12], investigated the effect of the addition of several metal carbides to SiC ceramics on their properties. The authors found that the thermal, electrical, and mechanical characteristics could be tuned over a quite wide range via the addition of relevant carbides, such as B_4_C, ZrC, NbC, and VC.

Most transition metal carbides exhibit outstanding properties, such as high hardness, high melting point, high thermal and mechanical stability, and resistance to chemical corrosion. Among them, VC seems to be one of the most suitable additives to SiC due to its low thermal conductivity and high hardness [13]. Both VC and β–SiC form face-centered cubic (FCC) lattices, but their lattice constants and bonding characters differ significantly; therefore, solid solubility is expected to be low. Spark plasma sintering technique establishes an entirely distinct set of conditions for the facilitation of this process. VC improves the sinterability of SiC composites more effectively than TiC, making it favorable for pressureless sintering. Furthermore, VC has been shown to match SiC more effectively in terms of thermal expansion, thereby contributing to a reduction in residual stresses. The effect of VC addition was investigated in ZrB_2_–SiC systems, and it was found that, in the combination of proper sintering conditions, significant improvement of mechanical characteristics could be obtained [14]. In comparison with the recently studied TiC [15], VC is less susceptible to grain growth in SiC ceramics during the sintering process, thus indicating the potential of achieving enhanced performance.

Vanadium carbide was chosen for the present study due to its superior grain growth inhibition efficiency and stability with SiC at high temperatures. The initial investigation on SiC–VC systems revealed that high density is achievable at sintering temperatures of 1900 and 2000 °C, reaching 91.2% for the 60SiC–40VC composite [16]. However, the effect of VC addition to SiC ceramics has still not been examined. Thus, the objective of the present study is to examine the impact of the vanadium carbide content, sintering temperature, and holding time on the hardness and fracture toughness of the SiC–VC composites. A novel methodology based on the two- and three-dimensional vector spaces of material features was applied for the description and analysis of the manufacturing process.

## 2. Materials and Methods

**Figure 1:**
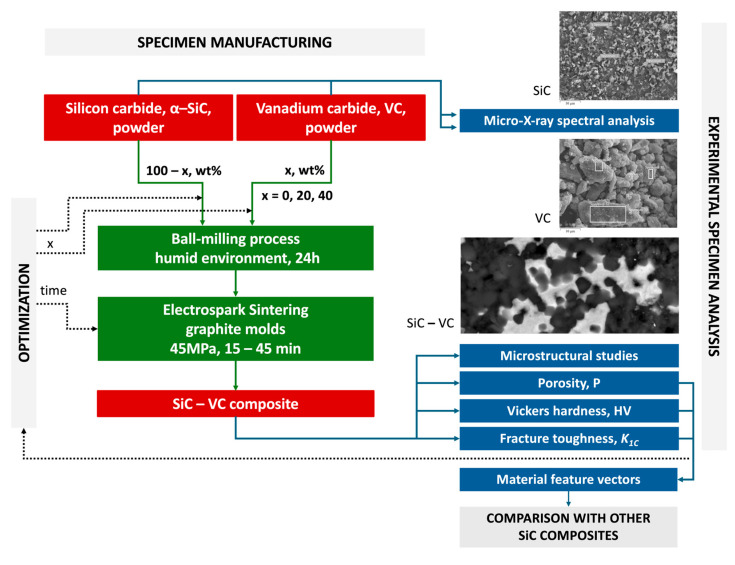
A flowchart of the research methodology.

A comprehensive flowchart illustrating the step-by-step methodology employed in the study is shown in Figure 1. It offers a visual representation of the stages and processes undertaken during the research.

Each element in the flowchart is representative of a discrete task, process, or decision point within the study, thereby providing an overview of the research methodology. This flowchart facilitates comprehension of the sequential progression of activities and methodologies implemented to achieve the objectives of the study.

### 2.1. Raw Materials

**Figure A1:**
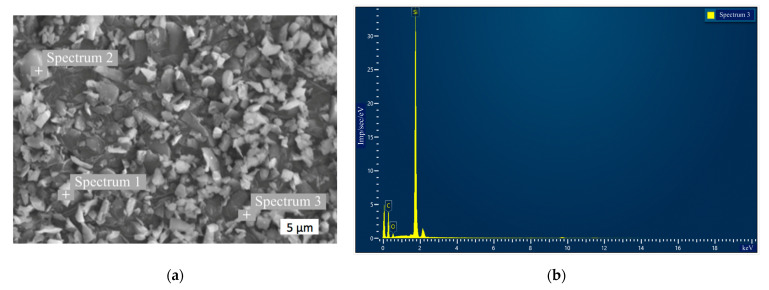
Energy-dispersive X-ray spectroscopy (EDS) of SiC powder: (**a**) SEM image with indicated spectra; (**b**) results for Spectrum 3.

For the purposes of this study, the powder α–SiC synthesized and delivered by the Zaporizhzhia Abrasive Plant PJSC (Zaporizhzhia, Ukraine) was used. It had an average particle size of 2 μm and comprised ~98% SiC, with no more than 0.1% Fe, 0.4% C, and 1.5% O. The results of energy-dispersive X-ray spectroscopy (EDS) for the initial SiC powder are presented in Appendix A, Figure A1, and Table A1. The Mahalanobis distances [17] suggest that all the data points fall within a certain range that is deemed typical. Methodology for the Mahalanobis distances is described in Appendix B, Appendix B.1.

**Figure A2:**
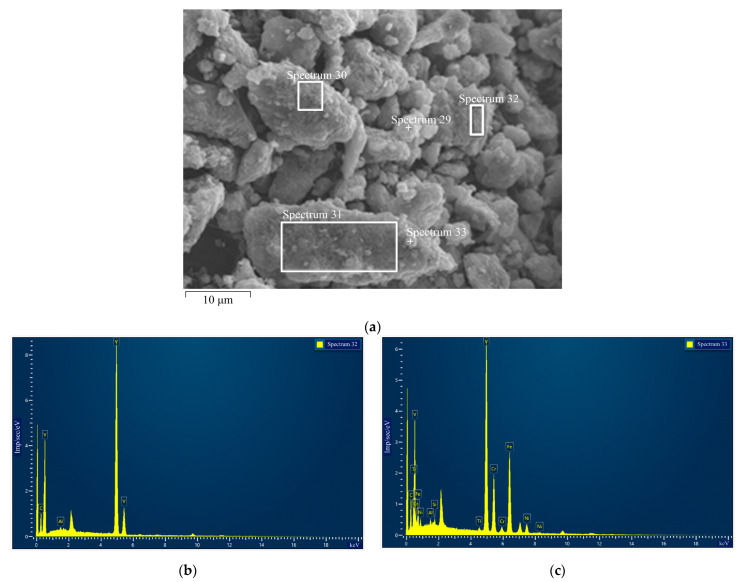
EDS results for VC initial powder: (**a**) SEM image with points chosen for analysis; (**b**) Spectrum 32; (**c**) Spectrum 33.

Vanadium (IV) carbide (VC) powder used as an additive exhibited an average particle size of 6 μm. The results of EDS analysis are presented in Appendix A, Figure A2, and the respective elemental percentages at each point are presented in Table A2. Notably, spectra from #29 to #32 looked almost identical, and only spectrum #33 revealed the presence of impurities.

The high concentration of iron, 30.72% in Spectrum 33 (the overall content of Fe should not exceed 0.1%), indicates that the impurities are unevenly distributed and tend to form agglomerates. However, the Mahalanobis distances suggest that there are no outliers, and Spectrum 30 is the most representative of all sets.

### 2.2. Material Processing

The initial powders were subjected to a ball-milling process for 24 h in a humid environment, with the application of grinding media composed of hot-pressed silicon carbide. For that purpose, the planetary ball mill Pulverisette 7 (Fritsch GmbH, Idar-Oberstein, Germany) was used. Samples consisting of SiC, SiC–20 wt.%VC, and SiC–40 wt.%VC were prepared. After that, the samples were sintered in graphite molds using a modified SPS apparatus. It was the original, patented device described in detail in [18]. The applied alternating current was 5000 A at a voltage of 5 V, which enabled the heating rate of 300 °C/min. The sintering process was carried out in a vacuum, ca. 1.5 Pa, at temperatures of 1900 °C and 2000 °C, under a uniaxial pressure of 45 MPa. Technically, the uniaxial pressure was applied after the temperature reached 1000 °C, then it was increased to 45 MPa after 3 min of subsequent heating, and 3 min after the start of cooling, it was dropped to 0 MPa. The samples were sintered using different holding times of 10, 30, and 45 min. The diameter of the sintered specimen was 11 mm.

### 2.3. Material Characterization

The material’s density and porosity were determined according to the methodology specified in the relevant regulations, DSTU EN ISO 3369:2014. Measurements of hardness HV were performed at a load of 150 N using a Matsuzawa MHT70 digital tester (produced by Matsuzawa Co., Ltd., Akita, Japan). The hardness and toughness were measured with *n* = 5 repetitions for each sample, and standard deviations were calculated as σ_HV_ ≈ 0.8–1.0 GPa. The pyramid indentations were studied using an NU–2E optical microscope (Carl Zeiss, Jena, Germany). The fracture toughness *K_1C_* was assessed using the commonly known Evans–Charles method [19], and respective standard deviations were calculated as σ_K1c_ ≈ 0.3–0.5 MPa·m^1/2^.

The material samples were examined at High Energy Technologies LLC using a Tescan Vega 3 SBH EP scanning electron microscope (produced by TESCAN, Brno, Czech Republic). The BSE (backscattered electrons) and SE (secondary electrons) modes were applied with an accelerating voltage of 20–30 kV. The elemental analysis was carried out with mapping methods at an accelerating voltage of 30 kV, scanning the surfaces of 0.09–0.25 mm^2^. The detection of elements ranging from B (Z = 4) to Am (Z = 95) was possible with a Bruker Quantax 610M EDS (energy dispersive X-ray spectroscope) (Bruker, Billerica, MA, USA). A description of the data processing methodology can be found in Appendix B.

The theoretical background of the study is provided in Appendix C, considering the sinterability of the SiC–VC system (Section Appendix C.1) and the thermodynamic perspective (Section Appendix C.2).

## 3. Results and Discussion

### 3.1. Densification and Porosity

**Figure 2:**
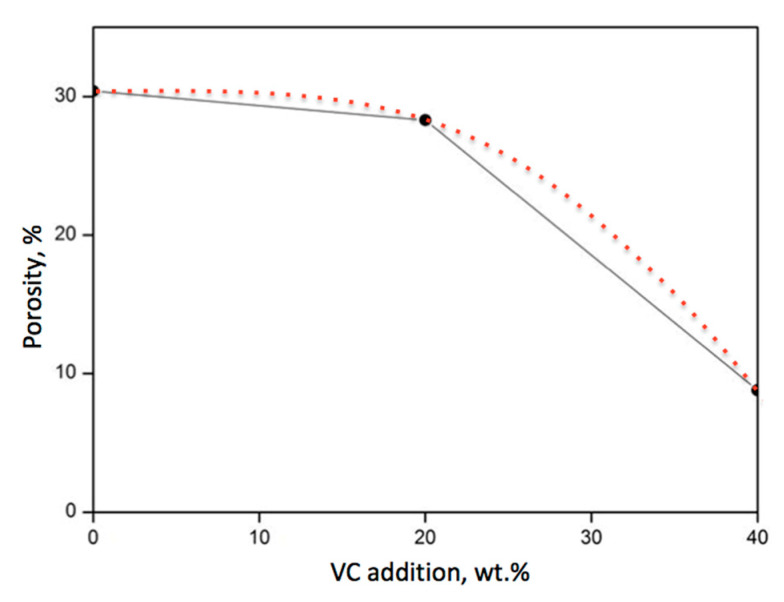
The dependence of the porosity of SiC-based composites on the addition of vanadium carbide. The sintering temperature was 2000 °C, and the holding time was 30 min.

It was expected that the addition of VC to the SiC powder would improve its densification. In this study, a very small improvement was observed after the addition of 20 wt.% VC. It remained at a level close to 30%, like in the case of pure SiC, as seen in Figure 2. However, when the proportion of VC was increased up to 40 wt.%, a significant reduction in porosity to 8.9% took place.

**Figure 3:**
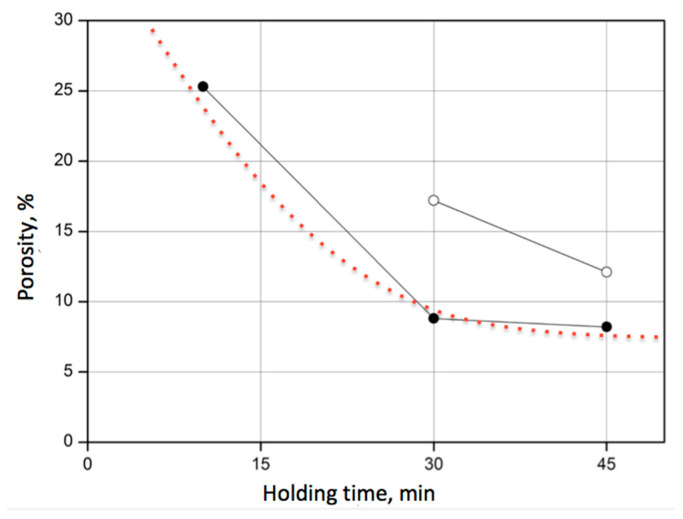
The dependence of the porosity of the 60SiC–40VC composite on the holding time at different sintering temperatures: 2000 °C (●); 1900 °C (○).

For the composite 60SiC–40VC, the effect of sintering temperature on the porosity was examined. As seen from Figure 3, reasonable densification with porosity below 30% could be achieved only after 10 min at a higher temperature of 2000 °C. A temperature of 1900 °C obtained the acceptable result of 17% only after 30 min. However, a lower sintering temperature did not ensure porosity below 10%, even after 45 min.

At the same time, it was found that the prolonged holding time at 2000 °C had an insignificant effect, especially considering the additional energy consumption. After 30 min, a porosity of 8.9% was reached, while after 45 min, it was 8.2%. Further prolongation of the holding time was found unreasonable.

### 3.2. Structure of SiC–VC Composites

**Figure 4:**
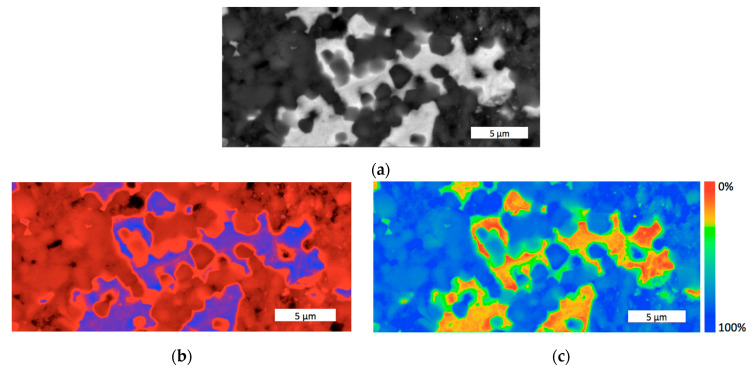
The microstructure of the 60SiC–40VC composite sintered at 2000 °C, with a holding time of 30 min: (**a**) SEM image; (**b**) phase contrast; (**c**) grain boundaries.

Figure 4a illustrates the microstructural features of the 60 wt.%SiC–40 wt.%VC composite, processed at a sintering temperature of 2000 °C, under a uniaxial pressure of 45 MPa, during the holding time of 30 min. The structure of the material consisted of gray grains of the silicon carbide matrix phase and light inclusions of vanadium carbide of dimensions mainly between 1 and 12 μm. The phase diagram shown in Figure 4b confirms the high purity of the VC reinforcement, while Figure 4c reveals the significant area covered by the grain boundaries. The color bar scale, ranging from red (0%), through yellow and green, to dark blue (100%), helps to enhance the discernibility of grain boundaries.

**Figure 5:**
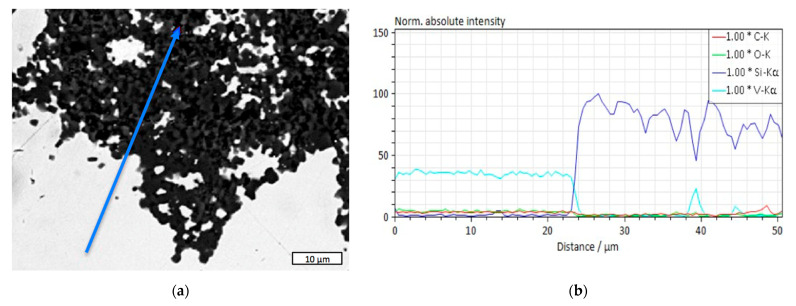
The interfacial zone between phases in the 60SiC–40VC composite: (**a**) the analyzed area with the sampling direction marked by the blue arrow; (**b**) M-XRF diagram.

The interfacial area between the matrix and the reinforcement in the composites plays a very important role. Figure 5 shows the micro-X-ray fluorescent spectroscopy (M-XRF) diagram providing an insight into the thickness of a boundary zone between SiC and VC. The intensity of the spectrum in Figure 5b along the blue arrow seen in Figure 5a indicates a clear stepwise change between the domination of vanadium in the distances between 0 and ca. 23 μm, and the area of silicon domination from ca. 24 μm to the end of the measurement axis. Thus, the interfacial layer exhibited a thickness of around 1 μm.

**Figure 6:**
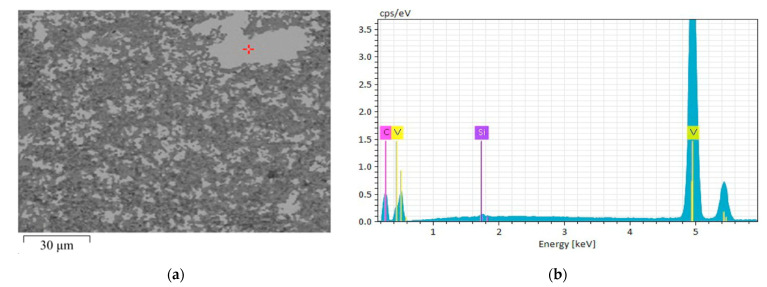
The energy-dispersive X-ray spectroscopy (EDS) qualitative results for the 60SiC–40VC composite sintered at 2000 °C, with a holding time of 30 min: (**a**) SEM image with the indication of the measurement point in a grey VC grain; (**b**) Spectrum 1804.

The elemental composition of the inclusions can be assessed from the EDS diagram shown in Figure 6. The SEM image in Figure 6a indicates the point in a large VC grain where the measurement was performed. From the diagram in Figure 6b, it can be seen that some amount of silicon was present in the grain of VC. The quantitative elemental composition is presented in Table 1, which reveals 0.23 wt.% of Si present in the analyzed VC grain. The general results of EDS analysis are collected in Table 2.

In general, it can be assumed that the sintered structure of the SiC–VC composite exhibited a high degree of homogeneity. Mainly, the near-micron VC inclusions acted as a particulate filler in the SiC matrix, with some exceptionally large grains like the one seen in Figure 6a. However, an important feature of the VC grains is the presence of the SiC inclusions inside. In the case seen in Figure 4a, some micrometer-sized SiC inclusions are observed in the VC grain of a dozen micrometers in size. This feature significantly increases the proportion and the role of interfacial areas in the composite.

### 3.3. Properties of SiC–VC Composites

**Figure 7:**
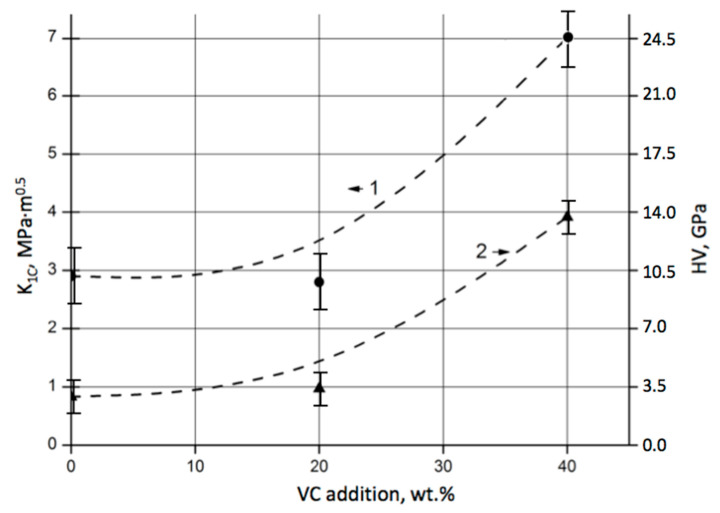
Toughness K_1C_ and hardness HV of SiC–VC composites with different vanadium carbide proportions sintered at 2000 °C, with a holding time of 30 min: 1—Fracture toughness K_1C_ (●); 2—Vickers hardness HV (▲).

The effect of VC addition on the physical and mechanical properties of SiC–VC composites can be seen from the diagram in Figure 7. It shows the dependence of the fracture toughness K_1C_ and Vickers hardness HV on the vanadium carbide content in SiC–VC composites sintered at a temperature of 2000 °C.

Again, like in the case of porosity discussed in Section 3.1, the addition of 20 wt.% VC had a very small effect on the toughness and hardness of the sintered composites. However, the 60SiC–40VC composition exhibited almost a 250% increase in K_1C_ and a 400% improvement in hardness HV.

The difference between the experimental data and the model at 20 wt.% VC can be explained by the insufficient volume of the VC-phase, which is unable to reach effective densification. The VC concentration was not enough to complete the network along the SiC grain boundaries. As a result, the porosity remained high, and the mechanical properties did not alter much from those of pure SiC. In that case, the model appeared to be sensitive to porosity, which did not correlate with the increase in hardness and toughness. When the content of VC reached 40 wt.%, the trends became more correlated. Moreover, at smaller amounts of VC in the SiC matrix, VC formed agglomerates, causing a local excessive presence. At 40 wt.%, VC was distributed more evenly in the matrix.

**Figure 8:**
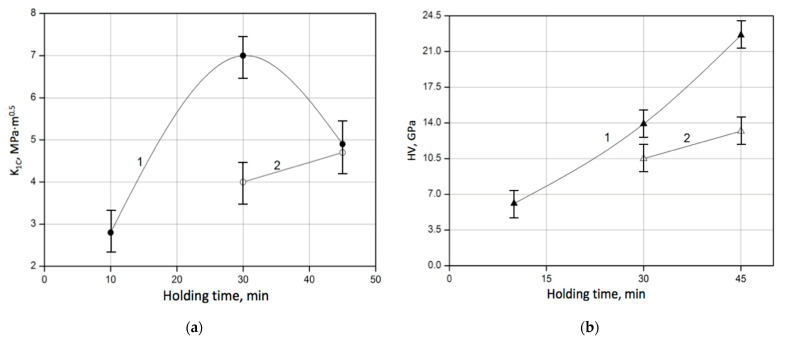
The dependences of the toughness and hardness of composites 60SiC–40VC on the holding time: (**a**) Fracture toughness K_1C_ reached 1 at a sintering temperature of 2000 °C (●); Fracture toughness K_1C_ reached 2 at a sintering temperature of 1900 °C (○); (**b**) Vickers hardness HV reached 1 at a sintering temperature of 2000 °C (▲); Vickers hardness HV reached 2 at a sintering temperature of 1900 °C (∆).

The dependence of toughness and hardness on the holding time is shown in Figure 8. The diagram in Figure 8a demonstrates that when the 60SiC–40VC composite was sintered at a temperature of 1900 °C for 30 min, the fracture toughness reached 4.0 MPa∙m^1/2^, and the hardness reached 10.5 GPa. However, when it was sintered at a temperature of 1900 °C for 45 min, the toughness increased up to 4.7 MPa∙m^1/2^, and the hardness reached 13.2 GPa. When sintering the composite at a temperature of 2000 °C and holding for 30 min, the fracture toughness increased to 7.0 MPa∙m^1/2^, and the hardness increased to 13.9 GPa. Extending the holding time to 45 min, it is possible to somewhat increase the hardness up to 22.6 GPa. At the same time, a decrease of 30% in the fracture toughness of the 60SiC–40VC material to 4.9 MPa∙m^1/2^ is observed. In our opinion, this effect can be attributed to the growth of silicon carbide grains that can lead to a decrease in grain boundary area, thereby reducing the opportunities for crack deflection and even leading to the appearance of brittle fracture paths. However, in small percentages, it can be caused by the carbon loss under vacuum. The carbon acts as an inhibitor of grain growth, so that in its absence, SiC grains are capable of growing abnormally [20,21].

A thorough analysis of the research data reveals that the addition of vanadium carbide to SiC up to 20 wt.% had an insignificant effect on the densification process, and the final values of porosity, fracture toughness, and hardness did not improve. However, a significant improvement was noted after the addition of 40 wt.% VC.

The increase of the sintering temperature of the 60SiC–40VC composite from 1900 °C to 2000 °C resulted in an improvement in hardness by ~70% from 13.2 to 22.6 GPa at a holding time of 45 min and of fracture toughness by ~50% from 4.0 to 7.0 MPa∙m^1/2^ at a holding time of 30 min. However, when the holding time was increased to 45 min, a drop in toughness by ~30%, accompanied by an increase in its hardness by ~60%, took place. The relationship between fracture toughness and holding time, in contrast to the relationship between hardness and holding time, appeared to be non-monotonic.

### 3.4. Material Feature Vectors

**Figure 9:**
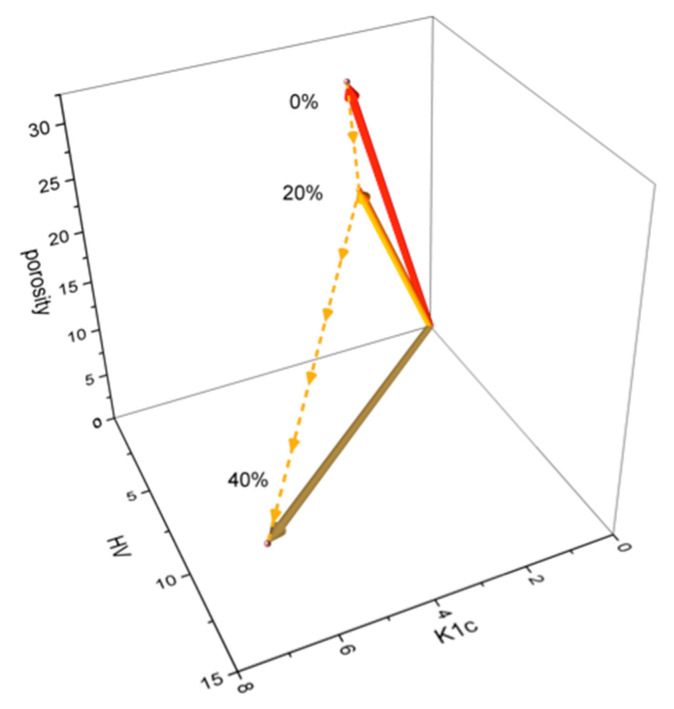
A visualization of the material feature vectors in the 3D material feature space.

Material feature vectors were found useful in describing the mechanical properties of the analyzed SiC–VC composites. As demonstrated in the analysis of the experimental data, it was evident that the combination of porosity, fracture toughness, and hardness was the underlying factor responsible for conferring the unique and highly sought-after properties of the sintered SiC–VC composites. The n-dimensional material feature vector, with each value corresponding to a specific property of the sample (K_1c_ and HV for *n* = 2, or porosity, K_1c_, and HV for *n* = 3, respectively), can be helpful in the characterization of the structure and mechanical properties of composites. The magnitude of the material feature vector and the angle between vectors, presented in Table 3 and Figure 9, highly depended on the VC proportion. Table 3 presents the 2D and 3D material feature vectors and angular distances (in degrees) between pure SiC (VC proportion 0%), SiC–20VC, and SiC–40VC. The margin of error was no greater than 4–5%.

As follows from the above data, the 3D material feature vector is much more sensitive to the VC proportion than the 2D material feature vector. Moreover, the angular distance between 20 wt.% and 40 wt.% is higher than that between 0 wt.% and 20 wt.%. The changes in the magnitude of the 2D and 3D material feature vectors listed in Table 3 have inverse monotonicity, which reveals the importance of the porosity parameter. However, the porosity of the material with the desired properties is usually determined with some uncertainty.

As it is well established in the relevant literature, composite materials can be obtained through a variety of methods, which, in turn, leads to significant fluctuations in their mechanical properties despite the same percentage of additives. Consequently, the prediction of the optimal percentage of the components required for the manufacturing of a composite material is a challenging task, and it should be related to the particular processing technology. The extent of the additive is closely related to the porosity. The precision of porosity estimation is contingent on the knowledge of the composite density.

**Figure 10:**
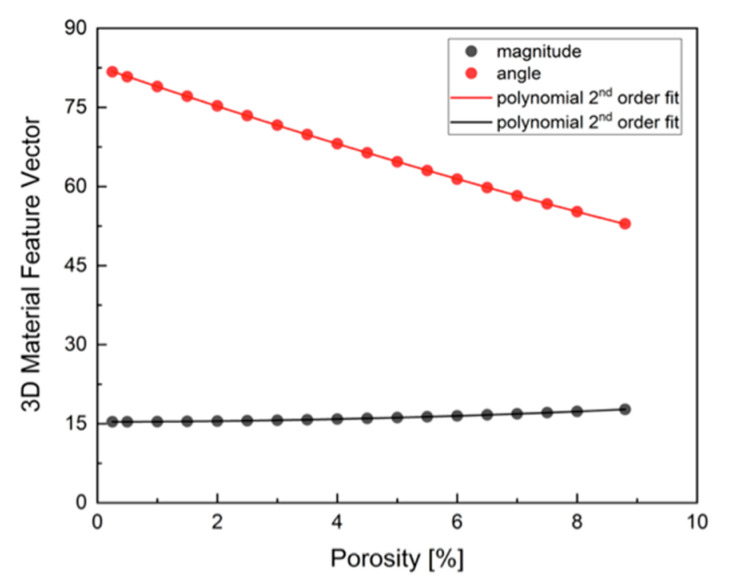
The influence of the porosity on the magnitude and angle of 3D material feature vectors.

An effort was made to model this effect and to estimate the potential influence of porosity uncertainty on the 3D material feature vector. The dependence of the magnitude and angle of the 3D feature vector on porosity is illustrated in Figure 10.

The diagram in Figure 10 was plotted under the assumption that porosity was the only factor affecting the material’s mechanical properties, i.e., the HV and K_1c_ parameters were fixed. It is imperative to note that this simplification had the potential to engender errors. Consequently, the porosity was modified solely within a circumscribed range. The effect of the porosity on both the magnitude and angle of the 3D material feature vector was found to be monotonic and was described using a second-order polynomial curve, with a Pearson coefficient of r = 0.999. As follows from Figure 9, the angle is more prone to variation than the magnitude when alterations in porosity are made.

In the context of recommendations of material properties, two material feature vectors should be close if the materials share common features. A comparison of their similarity estimated using Euclidean distance (ED) or cosine similarity (CS) can facilitate the estimation of the rate of change, which is advantageous for optimization processes. As the ED value increases, the discrepancy between vectors becomes more pronounced. Conversely, as the CS decreases, the similarity between vectors becomes more apparent.

The values of ED and CS between material feature vectors for 0, 20, and 40 wt.% of the VC additive are listed in Table 4 and Table 5, respectively. They are presented in form of a red–yellow–green scheme, where green indicates a maximum value and red indicates a minimum value.

The highest ED and the lowest CS between the 2D and 3D material feature vectors illustrate the progression of features resulting from alterations in the proportion. In addition, they are indicative of the optimal choice when considering material properties.

**Figure 11:**
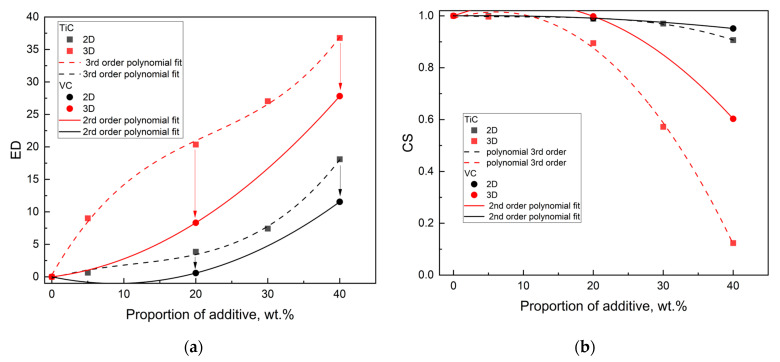
The similarity of the material feature vectors versus VC from this study and TiC [15] proportion: (**a**) Euclidean distance; (**b**) cosine similarity. The arrows denote the alterations from TiC to VC.

The behavior of the 2D and 3D material feature vectors for the SiC–VC was compared to that of SiC–TiC composites [15] since both were obtained by the same electroconsolidation technique. As the proportion of VC increases, the discrepancy between the ED values becomes more pronounced (Figure 11a), but the CS becomes more apparent (Figure 11b).

**Figure 12:**
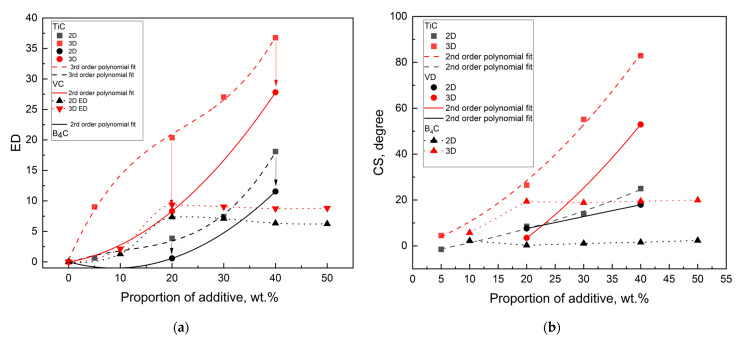
A comparison of the similarity of the material feature vectors for SiC–VC, SiC–TiC [15], and B_4_C–SiC [23]: (**a**) Euclidean distance; (**b**) CS-derived angular distance. The arrows denote the alterations from TiC to VC.

The behavior of the 2D and 3D material feature vectors for the SiC–VC, as well as SiC–TiC, composites obtained by electroconsolidation was monotonic. This finding indicates that the material properties of SiC–VC, similar to SiC–TiC, exhibited a smooth transition in response to VC proportion. The tendency for VC resembled that of TiC, albeit to a lesser extent. Thus, the material properties of SiC–VC, as illustrated in Figure 11, manifested a more nuanced transition in response to the VC additive when compared to that of TiC. As demonstrated in Figure 12, the dependencies of CS and ED on the proportions of the respective additives appear to indicate a comparable trend in the alteration of materials following a change from TiC to VC. The polynomial curves, which have been fitted to the experimental points, bear a resemblance in character. Furthermore, it is evident that the curves are approximately equidistant. However, a decrease in the ED and CS for VC compared to TiC is noticeable. This finding indicates that the SiC material reinforced with VC exhibited distinct characteristics when compared to the one with TiC.

From the perspective of material science, it is interesting to make a comparison with boron carbide B_4_C, a synthetic material characterized by its elevated level of hardness, frequently employed as a matrix in composites with SiC reinforcement [22,23]. In a SiC matrix with TiC added, Ti can substitute Si, and similarly, in SiC with VC addition, V can substitute Si. In contrast, when silicon carbide is added to B_4_C, B substitutes for C. Therefore, boron is used to aid densification, often in combination with carbon (B + C). Thus, boron has a very different effect on SiC transport properties compared to that of titanium or vanadium. It should be noted, however, that in many works on SPS sintering of SiC with B_4_C or B + C, the amount of additive is typically very small at 1–3%, which allows SiC to remain the dominant material. In our research, the addition of 40 wt.% VC significantly modified the structure of SiC, partially in the form of a solid solution between SiC and VC, and partially as a composite. We focus mostly on VC influence on densification, grain growth inhibition, and the related mechanical properties. Thus, the material can be regarded not only as SiC with an additive but rather as a SiC–VC composite system with modified structural characteristics.

Surprisingly, the character of the ED–proportion and CS-derived angular distance–proportion dependences for B_4_C–SiC [23] appeared to be similar to that of TiC–SiC [15] and VC–SiC. This is seen in Figure 12. Both exhibit a distinct, slightly declining plateau above 20 wt.%, resembling a sigmoid type curve with an inflection point at 10 wt.%.

**Figure 13:**
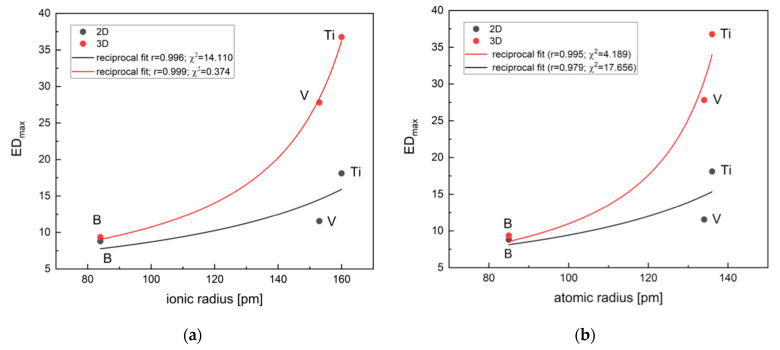
The 3D maximum values ED_max_ for the examined ceramics: (**a**) in relation to the ionic radius; (**b**) in relation to the atomic radius.

The inflection points for the ED–proportion relations for SiC–TiC and SiC–VC, shown in Figure 13, are located at 20 wt.%, indicating that a plateau can be expected at 40 wt.%. Thus, a proportion of VC and TiC equal to 40 wt.% should mark the beginning of the plateau and optimum value.

The 3D maximum values of ED (ED_max_) for ceramic material versus ionic and atomic radii of the transition metals are shown in Figure 13. The diagrams illustrate the Zener pressure pinning. The neglect of the porosity in the material feature vectors resulted in higher points scattering.

Vanadium possesses smaller ionic and atomic radii and higher electronegativity in comparison to titanium. Therefore, V has a lower affinity for carbon than Ti, and carbon–vanadium bonds are generally weaker than those of carbon–titanium. Consequently, the rate of carbon diffusion is expected to be higher in SiC–VC than in SiC–TiC. It is well known that carbon hinders the transport inside the material and inhibits SiC grain growth [21]. Thus, carbon addition results in partial densification of SiC by the removal of silica via carbothermal reduction. However, Table 1 and Table 2 suggest a nearly constant carbon level throughout the sintered sample, with a low silicon level and a high vanadium level in the inclusion area.

Vanadium added to SiC can also hinder transport due to the deep energy levels it introduces within the band gap (far from the conduction or valence bands), acting as carrier traps and recombination centers. This effect has the potential to exert a detrimental influence on material properties at elevated sintering temperatures, given that the driving force for grain boundary migration increases at such temperatures.

Since the atomic size of titanium is larger than that of vanadium, it is expected that titanium atoms would be rejected slightly more easily than vanadium ones during the electroconsolidation process. V atoms rejected from the lattice upon high-temperature exposure during sintering can form oxides more easily than Ti. Moreover, the violated vanadium oxide phase, V_2_O_3_, formed in the early stage of sintering due to the oxidation of VC, will apply pressure at the grain boundaries (Zener equation), reducing the grain growth and maintaining the smaller grain size. TiC is more susceptible to grain growth in sintering. Thus, VC acts as a microstructural enhancer and grain growth inhibitor, grain boundary phase or additive, while TiC acts rather as a sintering aid and toughening agent. In fact, both B_4_C and TiC act predominantly as densifiers. However, B is small and can easily substitute C sites, while the substitution of Si sites by Ti and V will introduce strain and consequently abnormal grain growth. Among the analyzed TiC, VC, and B_4_C, the titanium carbide seems a better balanced option for toughness and stability in SiC composites.

**Figure 14:**
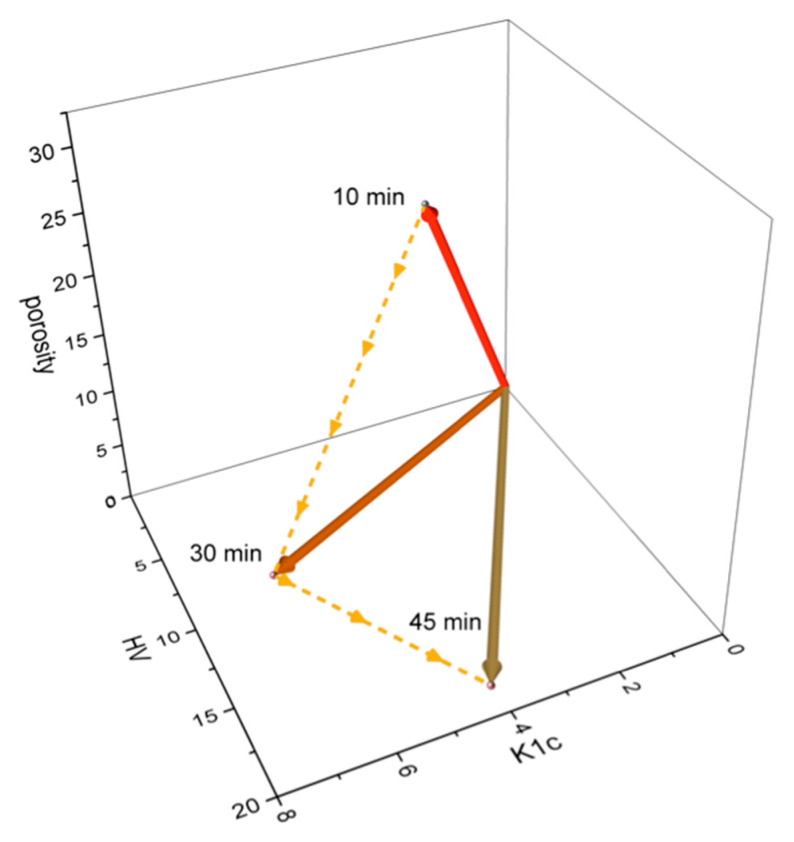
A visualization of the material feature vectors at 2000 °C in the 3D material feature space.

In order to assess the effect of the holding time at different sintering temperatures, the 2D and 3D material feature vectors and their angular distances (in degrees) were calculated and are collected in Table 6. The margin of error is below 5%. For the sintering temperature of 2000 °C, a visualization of the 3D material feature space is presented in Figure 14.

It should be noted that the material feature vector for the SiC–VC system with 40 wt.% VC sintered at T = 2000 °C with a holding time of 45 min exhibited the greatest divergence. The analysis of the material feature vectors’ similarity suggests that the 40 wt.% addition of VC and sintering temperature T = 2000 °C with 45 min of holding time can be the best option. However, the difference between the properties obtained after 30 min and 45 min is small, so that prolongation of the process is unprofitable considering the time and energy savings.

The values of the Mahalanobis distances (d_M_) determined for each observation are listed in Table 7. For the purpose of the comparative study, the SiC–TiC systems [15] were considered, too. To establish whether any of these distances are statistically significant, the corresponding *p*-values were calculated and added to the table.

Among the SiC ceramics with different analyzed additives (VC, TiC, and B_4_C), the material feature vector for SiC–VC with 40 wt.% of VC sintered at T = 2000 °C with a holding time of 45 min exhibited the greatest divergence from the others. It is notable that the Mahalanobis distance was the most significant for SiC–VC (d_M_ = 2.5096), yet it did not constitute an outlier at a 0.95 confidence level and a chi-square threshold of 7.815. The Mahalanobis distance reveals the distinctive nature of the SiC–VC composite.

The non-dominated vectors that form the Pareto front and represent the set of the best trade-offs between minimizing the first and maximizing the second and third components of the 3D material feature vectors also suggested choosing 40 wt.%, 2000 °C, and a holding time of 30 or 45 min. However, Pareto analysis favors 40 wt.% TiC over VC. Thus, all methods lead to similar decision-making conclusions.

It is worth noting that SiC properties render it particularly well-suited for medical applications, including those in dentistry, orthopedics, and cardiology, due to its proven biocompatibility and hemocompatibility. SiC appears to be an exceptional material, even for implants in the human brain and the treatment of deep tissue tumors because it does not induce an immune response. The comparative analysis reveals that the addition of VC instead of TiC resulted in enhanced fracture toughness, accompanied by reduced hardness of the SiC-based composite. This is advantageous for dental implant applications, as it prevents excessive pressure and damage to the tooth surfaces in contact with the implant. However, the addition of VC to SiC necessitates appropriate encapsulation due to the possible release of toxic vanadium ions by VC. Thus, the newly obtained SiC–VC system has potential, especially in surface engineering, structural applications, high-temperature use, and biosensors, but it requires additional studies to become an alternative to SiC–TiC composites. Vanadium ion toxicity and poorly tested long-term biocompatibility are its current limitations.

## 4. Conclusions

From the research results, it can be concluded that the presence of the reinforcing VC particles in the SiC matrix contributed to the reduction of porosity and improvement of fracture toughness. However, the effect is significant for 40 wt.%, while the addition of 20 wt.% of VC to SiC caused insignificant enhancement. It was also found that an increase in the sintering temperature of the 60SiC–40VC composite from 1900 °C to 2000 °C caused an improvement in hardness by ~70% and in fracture toughness by ~50%.

The Euclidean distance, cosine similarity, Mahalanobis distance, and Pareto analyses of the 3D material feature vectors led to similar decision-making conclusions. They indicate that the 40 wt.% addition of VC, 2000 °C, and 45 min of holding time are the parameters resulting in the best mechanical properties. However, considering the very small difference between the characteristics obtained after 30 and 45 min, the latter seems unprofitable due to the additional processing time and related energy consumption.

The comparative analysis reveals that the SiC-based composite with the addition of VC instead of TiC exhibited enhanced fracture toughness, accompanied by reduced hardness. This is advantageous for dental implant applications, but vanadium ion toxicity and poorly tested long-term biocompatibility are the current limitations and require further research.

## Figures and Tables

**Table 1:** The results of EDS quantitative analysis in the VC grain (Spectrum 1804).

Element	At. No.	Net	Mass, %	Mass Norm., %	Atom, %	Comp.	St., %	St. Norm., %	Abs. Error, % (1 Sigma)	Rel. Error, % (1 Sigma)
C	6	4110	18.33	19.12	50.00	–	0.00	0.00	3.04	16.57
Si	14	564	0.23	0.24	0.27	SiC	0.33	0.35	0.05	21.81
V	23	111,837	77.32	80.64	49.73	VC	95.55	99.65	2.14	2.77
Total	–	–	95.88	100.00	100.00	–	95.88	100.00	–	–

**Table 2:** The results of general EDS analysis (Spectrum 1805).

Element	At. No.	Net	Mass, %	Mass Norm., %	Atom, %	Comp.	St., %	St. Norm., %	Abs. Error, % (1 Sigma)	Rel. Error, % (1 Sigma)
C	6	964	21.34	21.96	50.00	–	0.00	0.00	5.03	23.55
Si	14	42,357	18.02	18.54	18.05	SiC	25.72	26.47	0.80	4.46
V	23	76,482	57.83	59.50	31.95	VC	71.47	73.53	1.62	2.80
Total	–	–	97.19	100.00	100.00	–	97.19	100.00	–	–

**Table 3:** The 2D and 3D material feature vectors and angular distance between pure SiC (VC content 0%), SiC–20VC, and SiC–40VC composites.

VC Proportion, wt.%	2D [K1C, HV]		3D [Porosity, K1C, HV]	
	Magnitude	Angle	Magnitude	Angle
0	4.40	—	32.30	—
20	4.28	7.59°	22.41	3.52°
40	15.38	17.94°	17.72	52.93°

**Table 4:** The Euclidean distance (ED) between the material feature vectors versus the VC proportion.

2D ED	0	20	40	3D ED	0	20	40
0	0	0.58	11.55	0	0	8.32	26.91
20	0.58	0	11.20	20	8.32	0	19.53
40	11.55	11.20	0	40	26.91	19.53	0

Note: Green indicates the maximum value, and red indicates the minimum value.

**Table 5:** The cosine similarity (CS) between the material feature vectors versus the VC proportion.

2D CS	0	20	40	3D CS	0	20	40
0	1	0.9912	0.9514	0	1	0.9981	0.6028
20	0.9912	1	0.9838	20	0.9981	1	0.6505
40	0.9514	0.9838	1	40	0.6028	0.6505	1

Note: Green indicates the maximum value, and red indicates the minimum value.

**Table 6:** The influence of the holding time on the 2D and 3D material feature vectors and their angular distances.

Holding Time, min	2000 °C				1900 °C			
	2D [K_1C_, HV]		3D [Porosity, K_1C_, HV]		2D [K_1C_, HV]		3D [Porosity, K_1C_, HV]	
	Magnitude	Angle	Magnitude	Angle	Magnitude	Angle	Magnitude	Angle
10	6.78	–	26.15	–	–	–	–	–
30	15.65	2.05°	17.95	45.64°	11.31	–	20.56	–
45	23.21	64.95°	24.62	35.30°	14.09	1.25°	18.58	15.99°

**Table 7:** The Mahalanobis distances and the respective *p*-values for TiC and VC additives.

Parameter	Additive	d_M_	*p*-Value
0 wt.%	–	1.6051	0.6582
5 wt.%	TiC	0.9565	0.8118
20 wt.%	TiC	1.7158	0.6334
30 wt.%	TiC	1.8918	0.5952
40 wt.%	TiC	1.9350	0.5860
20 wt.%	VC	1.5137	0.6791
40 wt.%	VC	2.1558	0.5407
40 wt.%, 10 min, 2000 °C	VC	1.1760	0.7588
40 wt.%, 30 min, 2000 °C	VC	2.1365	0.5446
40 wt.%, 45 min, 2000 °C	VC	2.5096	0.4736
40 wt.%, 30 min, 1900 °C	VC	0.4697	0.9255
40 wt.%, 45 min, 1900 °C	VC	0.5099	0.9167

## Data Availability

The original contributions presented in this study are included in the article. Further inquiries can be directed to the corresponding authors.

## Appendix A

**Table A1:** Elemental proportions of SiC powder.

Element	Spectrum 1, %	Spectrum 2, %	Spectrum 3, %	Max	Min	Average	Standard Deviation
C	54.99	59.30	59.94	59.94	54.99	58.08	2.69
O	6.34	4.58	4.17	6.34	4.17	5.03	1.15
Si	38.67	36.12	35.89	38.67	35.89	36.90	1.54
Total	100.00	100.00	100.00	–	–	–	–
Mahalanobis distance	1.954	1.139	1.138	–	–	–	–

**Table A2:** Elemental proportions of initial VC powder.

Element	Spectrum 29, %	Spectrum 30, %	Spectrum 31, %	Spectrum 32, %	Spectrum 33, %	Max	Min	Average	Standard Deviation
C	14.43	10.26	7.46	11.92	12.50	14.43	7.46	11.31	2.62
Al	0.83	0.57	0.46	0.62	0.65	0.83	0.46	0.63	0.13
Si	–	–	–	–	0.42	0.42	0.00	–	–
Ti	0.58	–	–	–	0.59	0.59	0.00	–	–
V	80.32	89.17	92.08	87.47	42.14	92.08	42.14	78.24	20.64
Cr	1.01	–	–	–	7.84	7.84	0.00	–	–
Fe	2.84	–	–	–	30.72	30.72	0.00	–	–
Ni	–	–	–	–	5.13	5.13	0.00	–	–
Total	100.00	100.00	100.00	100.00	100.00	–	–	–	–
Mahalanobis distance	1.784	0.651	1.649	1.573	1.789	-	-	-	-

## Appendix B. Data Processing

### Appendix B.1. The Mahalanobis Distance

The Mahalanobis distance (MD) is the statistical distance proposed in [17]. It quantifies the dissimilarity between two data points in a multi-dimensional feature space, considering the statistical fluctuation of each component. The MD is a multivariate generalization of the square of the standard score $\frac{x-\mu }{\sigma }$, which quantifies how a point differs from the mean of the probability distribution μ, expressed in units of standard deviation σ.

The MD can be calculated using the following formula:

(A1) $$d_{M}={\left({\left(p-\mu \right)}^{T}C^{-1}(p-\mu )\right)}^{1/2},$$

where p is a point, μ is a mean in n-dimensional space, and C is a semi-defined positive covariance matrix.

In consideration of the data distribution, the MD is capable of both identifying anomalous data points within their respective contexts and determining the points that are most representative. The uncertainty value of d_M_ can be derived using the absolute value of the total differential. The MD approximates a chi-square distribution with a degree of p′. Therefore, if d_M_ > χ^2^(p′)(1 − α), where α is a confidence level, then the sample is an outlier. However, the Mahalanobis distance choice is not optimal because large uncertainties, i.e., large covariance matrices, can lead to very small distances.

### Appendix B.2. Material Feature Vector Approach

In the report on previous research [15], we introduced a new approach, based on the n-dimensional feature vectors in an n-dimensional feature vector space, as a convenient tool for material characterization and optimization. The material feature vectors combine the parameters required for the assessment of material quality (i.e., grain size, hardness, and fracture toughness) into a convenient vectorial form useful for further comparative studies. The angles and magnitudes that describe these vectors are directly related to the materials’ properties. Consequently, the comparison of the properties of the materials can be reduced to a comparison of the properties of vectors.

The cosine similarity (CS) between the material feature vectors is defined as follows:

(A2) $$d_{c}\left(p,q\right)=\frac{\sum_{i}p_{i}q_{i}}{{\left(\sum_{i}p_{i}^{2}\sum_{i}q_{i}^{2}\right)}^{1/2}},$$

where <p> = {p_i_} and <q> = {q_i_} are the material feature vectors. The uncertainty value of d_c_(p, q) can be derived using the following equation:

(A3) $$\left|∆d_{c}\left(p,q\right)\right|=\sum_{i}\frac{q_{i}\sum_{j\neq i}p_{j}^{2}-p_{i}\sum_{j\neq i}p_{j}q_{j}}{{\left(\sum_{i}p_{i}^{2}\right)}^{3/2}{\left(\sum_{i}q_{i}^{2}\right)}^{1/2}}\left|∆p_{i}\right|+\sum_{i}\frac{p_{i}\sum_{j\neq i}q_{j}^{2}-q_{i}\sum_{j\neq i}p_{j}q_{j}}{{\left(\sum_{i}p_{i}^{2}\right)}^{3/2}{\left(\sum_{i}q_{i}^{2}\right)}^{1/2}}\left|∆q_{i}\right|,$$

where <Δp> = {Δp_i_} and <Δq> = {Δq_i_} are the error vectors.

CS is useful for the comparison of the vectors based on their directional similarity, but differences in magnitude between them are omitted. The Euclidean distance (ED) between the material feature vectors can be defined using the Euclidean metric, L2 norm, as follows:

(A4) $$d_{E}\left(p,q\right)={\left(\sum_{i}{\left(p_{i}-q_{i}\right)}^{2}\right)}^{1/2},$$

where <p> = {p_i_} and <q> = {q_i_} are the material feature vectors. The uncertainty value of d_E_(p, q) can be derived using the formula:

(A5) $$\left|∆d_{E}\left(p,q\right)\right|=2|\sum_{i}\left(p_{i}-q_{i}\right)|(\left|∆p_{i}\right|+\left|∆q_{i}\right|),$$

where <Δp> = {Δp_i_} and <Δq>{Δq_i_} are the error vectors.

### Appendix B.3. Pareto Multi-Objective Optimization of Material Feature Vectors

Pareto’s methodology is employed in the context of multi-objective optimization problems, wherein there exist numerous conflicting objectives that require simultaneous optimization. Rather than identifying a single optimum solution, a set of trade-off solutions is identified, termed the Pareto front, where the enhancement of one objective necessarily results in the deterioration of at least one other.

In the context of a set of material feature vectors from an n-dimensional material feature space representing multiple objectives, a vector is deemed to be Pareto optimal if no other vector in the set dominates it. The dominance of vector A over vector B is characterized by the superiority of A in all components, with at least one additional component exhibiting a higher performance rating. The collection of all Pareto optimal vectors forms the Pareto front, which represents the set of optimal trade-offs.

Consider a set of material feature vectors where the components represent an objective to be minimized (porosity), and objectives to be maximized (toughness K_1C_ and hardness HV). A vector is Pareto optimal if no other vector exists that has a smaller or equal porosity and simultaneously greater than or equal K_1C_ and HV components, with at least one strict inequality in one of the three objectives. The set of all such non-dominated vectors constitutes the Pareto front, representing the optimal trade-offs when minimizing the porosity and maximizing K_1C_ and HV. When selecting solutions that optimally balance competing criteria, this approach can assist the decision-making process.

## Appendix C. Theoretical Background

### Appendix C.1. Sinterability of SiC–VC System

Silicon carbide (SiC) is a ceramic material that exhibits strong covalent bonding, high hardness, low thermal expansion, and excellent thermal and chemical stability. Its low self-diffusion coefficient and high covalent character make it difficult to perform proper densification of SiC through conventional sintering techniques. To enhance sinterability, additives such as transition metal carbides are commonly introduced.

Among them, vanadium carbide (VC) exhibits good wettability with SiC. Considering its high hardness and thermal stability, it is very effective in improving the densification of SiC-based materials. VC can form solid solutions with SiC, segregate at grain boundaries, and influence both densification kinetics and grain growth. Depending on its proportion, VC can act as a sintering aid, a grain growth inhibitor, and a secondary reinforcing phase of the composite.

During electroconsolidation, which is a modification of spark plasma sintering (SPS), the uniaxial pressure is combined with the alternating current, densifying powder compacts rapidly. The electrical current produces localized Joule heating, leading to enhanced diffusion rates and enabling densification at lower temperatures and shorter sintering times compared to conventional methods [24,25].

The densification rate under electroconsolidation can be described by a modified Arrhenius equation, as follows:

(A6) $$\frac{d\rho }{dt}=A{\left(\frac{P}{d}\right)}^{n}exp\left(\frac{-Q_{eff}}{RT}\right),$$

where ρ is relative density, A is the material- and system-specific constant, P is applied pressure, d is the particle diameter, n is the empirical exponent, typically 1–2, Q_eff_ is the effective activation energy, R is the universal gas constant, and T is the absolute temperature.

The presence of VC exerts a modifying influence on the structure of SiC, impacting grain boundary chemistry and phase formation processes. It may form (Si,V)C_x_ solid solutions at elevated temperatures, particularly under EC conditions where high local energy facilitates substitutional diffusion. At elevated temperatures, the increased atomic mobility facilitates the formation of (Si,V)C_x_ solid solutions, where the silicon and vanadium atoms randomly substitute each other within the carbon-containing lattice.

Grain growth suppression promoted by the VC admixture can be explained by the Zener pinning effect, described by the following equation:

(A7) $$P_{z}=\frac{3f\gamma }{2r}$$

where P_z_ is pinning pressure, f is the volume fraction of second-phase particles (VC), γ is the grain boundary energy, and r is the particle radius of the second phase.

The substantial pinning pressures are the result of two factors: an increase in the volume proportion of particles and a decrease in the size of the particles. Higher pinning forces result in grain boundaries being pinned by particles; conversely, low forces cause further grain growth. Zener pinning pressure is orientation-dependent, implying that its exact value is contingent on the degree of coherence present at the grain boundaries.

### Appendix C.2. Thermodynamic Considerations

Reactions during sintering, such as the formation of (Si,V)C_x_ or potential interaction between VC and SiO_2_ surface layers, can be thermodynamically evaluated using Gibbs free energy, as follows:

ΔG = ΔH − TΔS. (A8)

A negative ΔG at the process temperature indicates the reaction that is thermodynamically favorable. Thermodynamic modeling and CALPHAD-based assessments have shown the potential for solid solution formation and minor interfacial reactions in the Si–V–C system [26].

The combination of SiC and VC in electroconsolidation systems facilitates improved sinterability, controlled microstructural evolution, and enhanced mechanical properties. In the course of the process, the rapid localized heating and concurrent pressure application promote diffusion-assisted densification, while VC plays a dual role as both a sintering aid and a microstructural stabilizer.

In the case of SiC, additives such as Al_2_O_3_ and rare-earth oxides, as well as the co-addition of boron and carbon, are typically employed [27,28]. These systems achieve effective densification at 1850–2000 °C for oxide additives and over 2000 °C when using B and C [29]. In B_4_C, a material of worse sinterability than that of SiC, sintering is facilitated by liquid-phase additives like Al, Ti, Ni, and Fe, as well as Al_2_O_3_ and Y_2_O_3_ [30,31,32]. Transition metal borides and carbides (e.g., TiB_2_ and CrB_2_) are also used, typically in concentrations above 10 wt.% [33,34,35]. Some additives not only promote densification but also improve mechanical performance. For instance, the addition of Al_2_O_3_ and Y_2_O_3_ to SiC results in a bending strength of 625 MPa and a fracture toughness of 7.5 MPa·m^1^/^2^ [36,37], while Ni-doped TiB_2_ ceramics exhibit values of 670 MPa and 6.4 MPa·m^1^/^2^, respectively [38,39].

Despite these successes, further enhancement in properties may be realized through sintering without additives, which would eliminate residual secondary phases typically found at grain boundaries. However, achieving full densification without sintering aids remains challenging. The most important drawbacks include grain growth during prolonged sintering, high operational costs, and limitations in component size due to equipment constraints. However, with careful control of particle size, sintering atmosphere, and temperature profiles, it is possible to achieve high-density ceramics with minimized grain growth and superior structural integrity [40].
